# Importance of Altered Gene Expression of Metalloproteinases 2, 9, and 16 in Acute Myeloid Leukemia: Preliminary Study

**DOI:** 10.1155/2021/6697975

**Published:** 2021-05-06

**Authors:** Jacek Pietrzak, Marek Mirowski, Rafał Świechowski, Damian Wodziński, Agnieszka Wosiak, Katarzyna Michalska, Ewa Balcerczak

**Affiliations:** Department of Pharmaceutical Biochemistry and Molecular Diagnostics, Laboratory of Molecular Diagnostics and Pharmacogenomics, Medical University of Lodz, Muszynskiego 1, Lodz 90-151, Poland

## Abstract

Acute myeloid leukemia is a group of hematological neoplasms characterized by a heterogeneous course and high mortality. The important factor in the neoplastic process is metalloproteinases, proteolytic enzymes capable of degrading various components of the extracellular matrix, which take an active part in modifying the functioning of the cell, including transformation to cancer cell. They interact with numerous signaling pathways responsible for the process of cell growth, proliferation, or apoptosis. In the present study, changes in the expression of *MMP*2, *MMP*9, and *MMP*16 genes between patients with AML and people without cancer were examined. The impact of cytogenetic changes in neoplastic cells on the expression level of *MMP*2, *MMP*9, and *MMP*16 was also assessed, as well as the impact of the altered expression on the effectiveness of the first cycle of remission-inducing therapy. To evaluate the expression of all studied genes *MMP*2, *MMP*9, and *MMP*16, SYBR Green-based real-time PCR method was used; the reference gene was *GAPDH*. For two investigated genes *MMP*2 and *MMP*16, the lower expression level was observed in patients with AML when compared to healthy people. The *MMP*9 gene expression level did not differ between patients with AML and healthy individuals which may indicate a different regulation of gene expression in acute myeloid leukemia. However, no correlation was observed between the genes' expression of all tested metalloproteinases and the result of cytoreductive treatment or the presence of cytogenetic changes. The obtained results show that the expression of *MMP*2 and *MMP*16 genes is reduced while the expression of *MMP*9 is unchanged in patients with acute myeloid leukemia. This may indicate a different regulation of the expression of these genes, and possible disruptions in gene transcription or posttranscriptional mechanisms in the *MMP*2 and *MMP*16 genes, however, do not affect the level of *MMP*9 expression. Obtained results in AML patients are in contrary to various types of solid tumors where increased expression is usually observed.

## 1. Introduction

Acute myeloid leukemia (AML) is a heterogeneity group of disorders of haemopoietic progenitor cell. Clonal proliferation of leukemic cells leads to incorrect hematopoiesis causing severe infections, anemia, or bleeding episodes [1]. Most patients diagnosed with acute myeloid leukemia are over 65 years when the efficacy of treatment is very low; about 70% die within one year of diagnosis [2]. Patients are usually treated with a standard chemotherapy scheme based on cytarabine and anthracyclines [3, 4]. The process of leukemogenesis is based on a two-hit model. The first panel of mutations occurs in the genes regulating proliferation and survival (*FLT*3, *PTPN*11, *Ras*, and *ETV*6*/PDGFbR*) [5]. The second one is associated with genes responsible for differentiation and apoptosis (*RUNX*1*/RUNX*1*T*1, *PML/RARα*, *KMT*2*A*, *CEBPA*, and *CBF*) [6]. This group contains genes-encoding proteins that are surface receptors for cytokines and growth factors which are transcriptional or epigenetic regulators of the cell cycle or apoptosis. Most of them are required in normal hematopoiesis [7]. The various chromosomal abnormalities are associated with hematological hyperplasia pathogenesis. In AML, more than half of cases are characterized by the presence of chromosomal aberration that could lead to the formation of fusion genes, causing the development of cancer [8]. Many different elements participate in the development of cancer, but one of the most important ones is metalloproteinases (*MMPs*) [9–13], which play a role in various types of hematological hyperplasia [14, 15]. Metalloproteinases not only play a key role in physiological processes such as tissue remodeling and organogenesis but also are important in pathological conditions, like the regulation of inflammation or primarily in the development of cancer [16]. The metalloproteinases' general structure consists of three domains: prodomain, catalytic domain, and hemopexin domain, which allowed distinguishing six groups: collagenases, gelatinases, stromelysin, matrylisin, cell membrane-associated metalloproteinases, and others [17]. The regulation of final metalloproteinase activity takes place at many levels from gene transcription to the activation of secreted proenzymes [18]. Metalloproteinases, through their proteolytic properties, are involved in tissue remodeling by regulating the composition of the extracellular matrix (ECM) as well as its three-dimensional structure. The extracellular matrix is modeled dynamically and adapted to the structure or function of different types of tissues [19]. Cancer-associated ECM is not only its integral component, but also a factor that actively influences various cellular mechanisms. Expression of genes involved in the processes of remodeling of extracellular matrix such as metalloproteinases or proteins that cause collagen cross-linking is a prognostic factor in cancer [20, 21]. The metalloproteinase promoter regions have several *cis* elements in their structure, enabling regulation by various transcription factors such as AP-1, ETV4, Sp1, *β*-catenin/Tcf-4, and NF-*κβ* [22]. In addition, many promoters for the genes of different metalloproteinases are identical, which leads to the coregulation of their expression [18]. Based on the arrangement of *cis* elements in the promoter region, metalloproteinases can be divided into three categories. The first group includes *MMP*1, *MMP*3, *MMP*7, *MMP*9, *MMP*10, *MMP*12, *MMP*13, *MMP*19, and *MMP*26, i.e., metalloproteinases, which have TATA sequences and sequences enabling binding to the transcription factor AP-1 in the promoter region [23]. The promoter regions of the second group of metalloproteinases *MMP*8, *MMP*11, and *MMP*21 also contain TATA sequences, while the AP-1 transcription factor binding site is missing [22]. The last group of the *MMP*2, *MMP*14, and *MMP*28 genes presents in promoters' regions lack of the TATA and AP-1. It is mainly determined by Sp1 transcription factors that bind to the GC region. Therefore, the expression of the *MMP*2, *MMP*14, *MMP*16, and *MMP*28 genes is primarily constitutive and only slightly regulated by other cytokines or growth factors [18, 24]. In the present study, changes in mRNA expression levels of selected metalloproteinase genes (*MMP*2, *MMP*9, and *MMP*16) in acute myeloid leukemia were evaluated. The majority of studies on the role of metalloproteinases in cancer development and progression have been conducted on solid tumors. Only several researches have focused on the function of metalloproteinases in hematological malignances. The conducted studies would be the basis for further analyses aimed at determining the role of metalloproteinases in acute myeloid leukemia. Obtaining potential differences would be the basis for conducting research on the direct effects of metalloproteinases on leukemic cells by silencing or overexpressing metalloproteinase genes. This knowledge could be applied to the use of inhibitors or metalloproteinases themselves as potential drugs in acute myeloid leukemia. The results of the study would provide information on the possible role of metalloproteinases in acute myeloid leukemia progression and impact on the result of chemotherapy treatment.

## 2. Materials

### 2.1. Materials

56 samples of whole blood taken from patients with acute myeloid leukemia and 60 blood samples from people without a cancer disease were analyzed. The study was approved by the Bioethical Commission of the Medical University of Lodz RNN/102/16KE and remained in accordance with the Declaration of Helsinki. The characteristics of AML patients are presented in Table 1. RNA was isolated from all samples.

## 3. Methods

### 3.1. RNA Isolation

RNA was isolated according to “Total RNA Mini Plus” protocol (A&A Biotechnology, Poland). Isolated RNA was transferred into tubes and stored at −80°C. Concentration and purity of the isolated RNA were assessed using a NanoPhotometer (Implen, Germany). The range of RNA concentration of the used samples was from 20.8 to 836 ng/*µ*l.

### 3.2. Reverse Transcription Reaction

After measuring the RNA amount, all samples were unified to identical, final concentration 0.05 *µ*g/*µ*l by adding appropriate amounts of water. Following that, reverse transcriptase-PCR was performed using High-Capacity cDNA Reverse Transcription Kits (Applied Biosystems, USA) according to the attached protocol. The reaction mixture contained 2.0 *µ*L 10 × RT Buffer, 0.8 *µ*l 25 × dNTP Mix 100 mM, 2.0 *µ*l oligo (dT), 0.5 *µ*g/*µ*l, 1.0 *µ*l, MultiScribe™ Reverse Transcriptase 20 U/*µ*l, 1.0 *µ*l RNase Inhibitor, and 20 U/*µ*l and 13.2 *µ*l RNA samples. After that, RT-PCR samples were stored at −20°C until further analysis.

### 3.3. PCR

PCR was performed for the *GAPDH* (housekeeping gene) to confirm the presence of an undegraded cDNA molecule. After that, a qualitative PCR for investigated genes was performed using the set of primers, whose sequences are presented in Table 2.

The PCR was performed using the JumpStart™ Taq DNA Polymerase without MgCl2 reagent kit (Merck, Germany). The reaction mixture consisted of 3.5 *µ*l 10x PCR buffer, 0.7 *µ*l magnesium chloride 25 mM, 0.4 *µ*l deoxynucleotide mix 10 mM, 0.2 *µ*L JumpStart Taq DNA Polymerase 0.05 U/*µ*l, 12.8 *µ*l sterile water, 0.7 *µ*l primer R for the tested genes 10 *µ*M, 0.7 *µ*l primer F for the tested genes 10 *µ*M, and 1 *µ*l cDNA template.

The reaction conditions were as follows: initial denaturation of 94°C for 1 min; denaturation at 94°C for 30 sec; annealing—*GAPDH*: 58°C for 30 sec, *MMP*2*:* 57°C for 30 sec, *MMP*9*:* 57°C for 30 sec, *MMP16:* 57°C for 30 sec; and elongation 72°C for 60 sec; the steps from the denaturation to elongation were repeated 34 times; final elongation 72°C for 5 min.

To confirm the presence of PCR products, all samples were separated into 2% agarose gel.

### 3.4. qPCR

**Figure 1:**
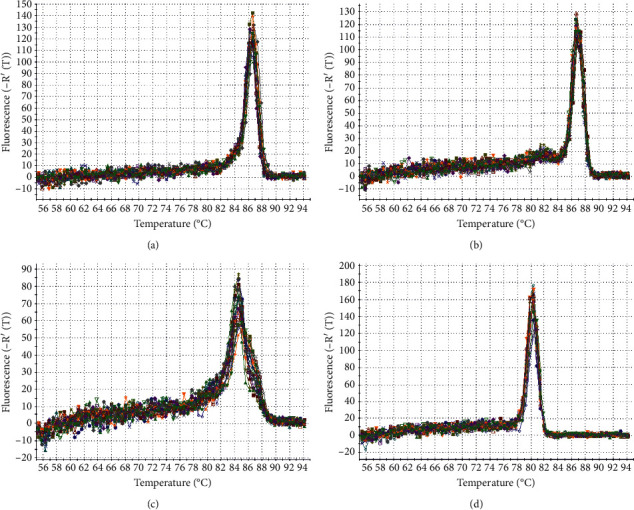
Melting plots for real-time PCR products. (a) GAPDH. (b) *MMP*2. (c) *MMP*9. (d) *MMP*16.

The analysis of all samples was performed in triplicate in the Stratagene Mx3000P analyzer (Agilent Technologies, Germany). The negative control without cDNA was added to each experiment. For the qPCR, the same sets of primers as for qualitative reaction were used (Table 2). The reaction mixture included 10 *µ*l 2x Bimake™ SYBR Green Master Mix (Bimake, USA), 0.4 *µ*l 50x ROX Reference Dye 2, 0.7 *µ*l primer R for the tested gene the concentration 10 *µ*M, 0.7 *µ*l primer F for the tested gene at the concentration 10 *µ*M, 1 *µ*l of cDNA tested sample, and 7.2 *µ*l of water. The reaction conditions were as follows: initial denaturation of 95°C for 10 min; denaturation at 95°C for 30 sec; annealing—for all tested genes (*GAPDH*, *MMP*2*, MMP*9, and *MMP*16): 58°C for 60 sec; elongation 72°C for 60 sec; the steps from the denaturation to elongation were repeated 40 times. Next to confirm the homogeneity of the obtained product, a thermal denaturation was carried out to obtain the melting curves (Figure 1). Quantitative analysis was performed in the next step, the first stage of which was to determine the efficiency of the qPCR for individual genes. The obtained results were *GAPDH*—100%, *MMP*2—93%, *MMP*9*—*99%, and *MMP*16—101.3%, respectively. The efficiency of reactions differed significantly; therefore, the Pfaffl method was used to calculate the relative expression ratio.

### 3.5. Statistical Analysis

The statistical analysis was performed using Statistica 13.1 (StatSoft, Inc., Tulsa, OK, USA). In all statistical tests used, the significance level was 0.05. The following statistical tests were used: Shapiro–Wilk test, Student's *t*-test, Mann–Whitney *U* test, and correlation matrices.

## 4. Results and Discussion

### 4.1. The Analysis of the Presence of Metalloproteinase Gene Expression

The presence of mRNA expression by qualitative PCR was confirmed for all investigated metalloproteinase genes (*MMP*2, *MMP*9, and *MMP*16) in the group of patients with AML and also in the control group.

### 4.2. Quantitative Analysis of the Expression of Metalloproteinase Genes in Patients with AML and in the Control Group

**Figure 2:**
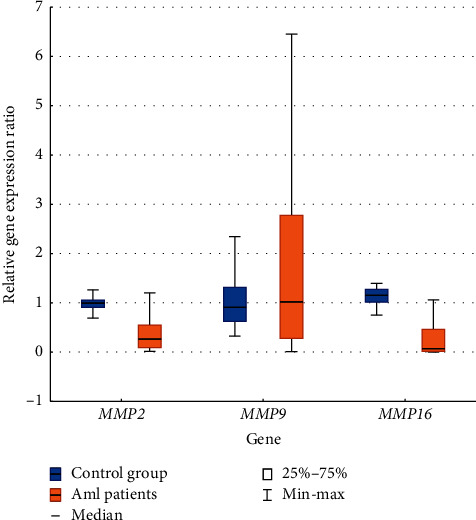
The relative expression levels of *MMP*2, *MMP*9, and *MMP*16 genes in the AML/control group.

After that, quantitative analysis was performed in both groups. First, the relative level of *MMP*2, *MMP*9, and *MMP*16 gene expression was compared between the control group and patients with AML. The statistical analysis revealed a significantly lower level of *MMP*2 (*p* < 0.0001) and *MMP*16 (*p* < 0.0001) gene expression in AML patients when compared to the control group. For *MMP* 9, such a difference was not observed (*p*=0.5723) (Figure 2).

**Figure 3:**
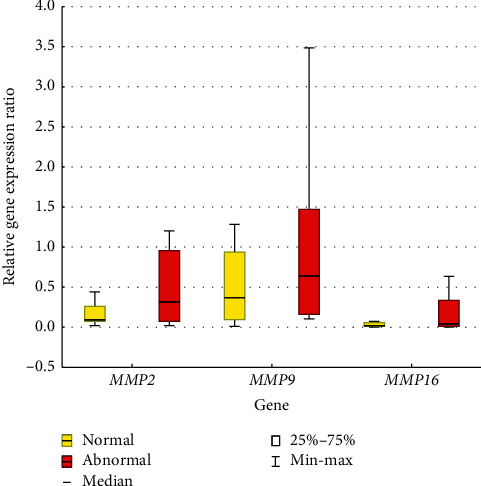
The relative expression level of *MMP*2, *MMP*9, and *MMP*16 genes in AML with normal/abnormal karyotype.

Subsequently, AML patients were divided into two subgroups, with various types of chromosomal abnormalities and with normal karyotype. The statistical analysis did not show any statistically significant correlation between the relative expression level of the *MMP*2 (*p*=0.4767), *MMP*9 (*p*=0.4212), and *MMP*16 (*p*=0.5982) genes and the presence of karyotype abnormalities (Figure 3).

**Figure 4:**
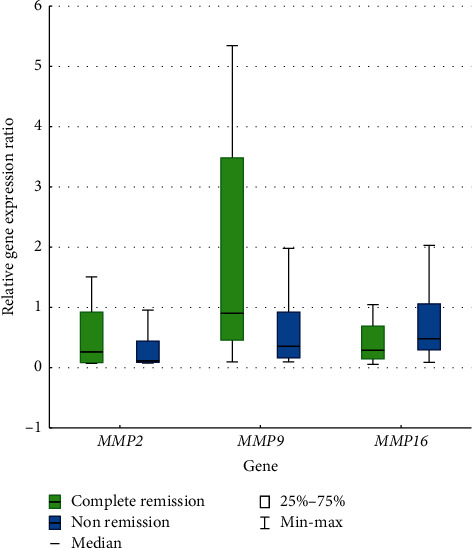
The relative expression level of *MMP*2, *MMP*9, and *MMP*16 genes in AML with complete remission/no remission after the first induction therapy.

The analogous analysis was carried out for checking a possible correlation between the expression levels of investigated genes and the effectiveness of induction therapy based on the three-day one-anthracycline and seven-day cytarabine regimen. The first group consisted of patients in whom the first cycle of treatment led to remission; the second one included patients in whom this scheme was not effective. There was no statistically significant difference in the expression levels of the *MMP*2 (*p*=0.9825), *MMP*9 (*p*=0.9076), and *MMP*16 (*p*=0.5617) and the result of chemotherapy (Figure 4).

In the group of patients with AML, the statistical analysis did not show any statistically significant difference between relative gene expression levels and gender (*MMP*2 *p*=0.5582; *MMP*9 *p*=0.5062; *MMP*16 *p*=0.3960). Similarly, no difference was found between the age at the moment of diagnosis of patients with acute myeloid leukemia and the relative expression level of *MMP*2 (*p*=0.298), *MMP9* (*p*=0.318), and *MMP*16 (*p*=0.753) genes.

The role of metalloproteinases in the development of solid tumors seems to be crucial. However, relatively few studies concern the problem of metalloproteinases function in hematological cancers. The obtained results showed a decreased expression level of *MMP*2 and *MMP*16 genes in the group of patients with AML and an unchanged expression level of *MMP*9 when compared to people without cancer diseases. The results are in contrast to previous studies that clearly indicated that metalloproteinases are factors that usually favor the development of the disease and their overexpression is observed in the course of neoplastic disease [16, 25, 26]. However, some studies show that metalloproteinases can play a role in both favoring and inhibiting tumor growth. The fact that can confirm this hypothesis is that so far it has not been possible to introduce metalloproteinase inhibitors into routine therapy despite many years of research on them. Mice model with *MMP*8 deficiency showed a greater susceptibility to developing cancer than mice with a physiological *MMP*8 protein concentration. In addition, a decrease in *MMP*8 gene expression in normal cells promotes the metastatic potential [27]. However, a better prognosis for patients with breast or oral cancer is correlated with a higher concentration of *MMP*8 protein [28]. *MMP*9 also has inhibitory and cancer-promoting effects. Many studies showed that *MMP*9 expression correlates with the clinical stage of cancer [29, 30]. Immunohistochemistry in breast cancer showed an association between the increased pro*MMP*9 and *MMP*9 and a shorter event-free survival [31]. However, studies, which also included early-stage breast cancer patients and examined only an inactive form, showed that the overexpression of *MMP*9 is associated with a longer event-free survival [32]. *MMP*16 is another example of inhibitory properties of metalloproteinases against cancer cells. Studies conducted on cell lines of the esophageal squamous cell carcinoma EC109 and EC9706 and tumor tissues showed a reduced expression of the *MMP*16 gene against normal tissue fragments. In addition, a decreased *MMP*16 expression correlated with the degree of lymph node involvement and a shorter patient survival. The induced overexpression of the *MMP*16 gene in EC109 and EC9706 cell lines resulted in arrest of cell division at the G1 phase stage [33]. According to the work of Ries et al. (1999), acute myeloid leukemia cells synthesize both *MMP*2 and *MMP*9 [34]. Measurements of *MMP*9 protein concentration in the bone marrow show lower levels in patients with AML than in the control group [35]. However, studies by Klein et al. also show that *MMP*9 levels in the bone marrow do not differ between healthy individuals and patients with AML [36]. A very similar situation also occurs in the case of *MMP*2. The studies of Kuittinen et al. show a positive correlation between the survival time of patients with acute myeloid leukemia and *MMP*2 concentration [37, 38]. However, Klein et al. observed an increased invasive potential of leukemia cells with the increased *MMP*2 production [36]. Studies on the role of *MMP*16 in hematological hyperplasia were conducted by Binato et al. only on stromal cells in myelodysplastic syndromes, which very often precedes the occurrence of AML and the results of this analysis show also a lower *MMP*16 gene expression in stromal cells in patients with myelodysplastic syndromes than in the control group [39]. A possible significant reason of the decrease in the expression of some metalloproteinases in the course of acute myeloid leukemia may be due to the impaired hematological cell function. Metalloproteinases play also an important role in the process of normal hematopoiesis, which has been confirmed by Yu and Han's studies [40], among others, which clearly indicated an increased concentration of metalloproteinases in the case of bone marrow renewal after its previous ablation. The function of *MMPs* in the process of hematopoiesis is pleiotropic and based primarily on the regulation between hematological cells and the bone marrow microenvironment which consists of extracellular matrix [40]. Extracellular matrix is a component in the development of acute myeloid leukemia, which is not only the structure that anchors the hematopoietic stem cells, but can also regulate functioning of cells by interacting with receptors belonging primarily to the integrin family [14]. *MMP*9 was the first metalloproteinase with confirmed effect on hematopoietic stem cells [41]. The ability of metalloproteinase 9 to cleave ligand for the c-Kit receptor from the surface of hematopoietic stem cells has also been demonstrated [14]. This receptor is expressed primarily on stem and progenitor cells. However, c-Kit receptor expression is reduced during cell differentiation. It is estimated that less than 1% of cells in peripheral blood express c-Kit, whereas acute myeloid leukemia cells are very often accompanied by overexpression of this receptor [42]. Another function of *MMP*9 in the course of normal hematopoiesis is participation in the regulatory axis CXCL12/CXCR4. The association of the CXCL12 ligand to the CXCR4 receptor is an important factor for the activation and migration of stem cells. *MMP*9, *MMP*2, but also *MMP*8 belonging to the collagenase group and *MMP*14 classified to the family of membrane metalloproteinases, similarly to *MMP*16, are able to inactivate CXCL12 by cleaving amino acids from the N-terminal end which are responsible for receptor binding [14]. In the case of acute myeloid leukemia cells, a variable number of CXCR4 receptors were observed on the cell surface; however, their number correlated with the migration potential of cancer cells. In addition, CXCR4 receptor inhibitors showed an inhibitory effect on the spread of leukemia cells [15]. Hematological stem cells have the ability to synthesize *MMP*2 and *MMP*9, which allows them to migrate through the basal membranes [43]. This effect seems to be more important during the implantation of cells after transplantation than for their activation. Stromal cells are also capable of synthesizing metalloproteinases. The movement of CD^34+^ cells through the layer of mesenchymal cells is conditioned by *MMP*2 which they produce [14]. The role of metalloproteinases in the functioning of acute myeloid leukemia cells may not only be determined by their synthesis by cancer cells but also by the synthesis of metalloproteinases by microenvironment cells, e.g., fibroblasts [34]. The problem with the determination of the metalloproteinases role in cancer may be due to the multistage regulation of their activity, which includes the process of gene transcription, posttranscriptional changes, the amount of secreted enzymes, and the impact on their activity of specific and nonspecific inhibitors. For many years, attempts have been made to design various types of metalloproteinase inhibitors that could be used in cancer therapy, for example, Marimastat or BAY 12-9566. However, none of them was included in routine therapy due to the discrepancy between preclinical and clinical trials [26].

## 5. Conclusions

The cells of people with acute myeloid leukemia have a lower relative gene expression level of the *MMP*2 and *MMP*16 but no *MMP*9 compared to patients without cancer. The expression level of the *MMP*2, *MMP*9, and *MMP*16 genes does not depend on the presence of cytogenetic changes in cancer cells and gender and age of patients with acute myeloid leukemia. Also, the mRNA level of *MMP*2, *MMP*9, and *MMP*16 genes at diagnosis does not determine the effectiveness of the remission-inducing treatment. The role of metalloproteinases in neoplastic disease, despite numerous evidences of significant participation in the development of the disease, may directly depend on the type of proliferation and its stage of advancement. Such a situation may result from numerous functions that are fulfilled by metalloproteinases, including participation in inhibiting the development of the tumor structure. For the final evaluation of the role of the studied metalloproteinases in the course of acute myeloid leukemia, it is necessary to perform tests based on their direct effect on cancer cells. For this purpose, it is planned to determine the effect of gene silencing and its overexpression on the proliferation of cancer cells and apoptosis.

## Figures and Tables

**Table 1:** Clinical characteristics of patients with acute myeloid leukemia.

Clinical feature	Types of cases	Number of cases (*N*)	Average age (years)
Gender	Men	25	57 (SD 14, 5)
	Women	31	60 (SD 15, 02)

AML morphological subtype	AML1	3 (2 W; 1 M)	59 (SD 13, 10)
	AML2	7 (5 W; 2 M)	61 (SD 12, 51)
	AML3	1 (1 M)	26 (SD 0, 00)
	AML4	6 (3 W; 3 M)	59 (SD 22, 94)
	AML5	3 (1 W; 2 M)	39 (SD 16, 21)
	AML6	1 (1 M)	57 (SD 0, 00)
	AML7	0	—
	Indefinite	35 (20 W; 15 M)	61 (SD 11, 51)

Karyotype	Normal	10 (3 W; 7 M)	55 (SD 9, 86)
	Abnormal	17 (10 W; 7 M)	56 (SD 16, 98)
	Indefinite	29 (18 W; 11 M)	63 (SD 10, 72)

Result of induction treatment	Complete remission	14 (6 W; 8 M)	48 (SD 14, 47)
	No remission	16 (7 W; 9 M)	63 (SD 9, 59)
	Indefinite	26 (18 W; 8 M)	64 (SD 10, 87)

W: women; M: men.

**Table 2:** Starter sequences of examined genes.

Gene	Starter type	Sequence from 5′ to 3′
*GAPDH*	Forward	ATG CCA GTG AGC TTC CCG TTC AGC
	Reverse	TGG TAT CGT GGA AGG ACT CAT GAC

*MMP*2	Forward	GTA TCT CCA GAA TTT GTC TCC
	Reverse	ATG AAT ACT GGA TCT ACT CAGC

*MMP*9	Forward	CGA GGA CCA TAG AGG TG
	Reverse	CTT AGA TCA TTC CTC AGT GC

*MMP*16	Forward	TCT GTC TCC CTT GAA ATA
	Reverse	ACC CTC ATG ACT TGA TAA CC

## Data Availability

The clinical data of patients used to support the findings of this study are included within the article. The data including gene expression in individual patients used to support the findings of this study are available from the corresponding author upon request.
